# Multidisciplinary Dental Rehabilitation in an Adult with Noonan Syndrome: A Case Report

**DOI:** 10.7759/cureus.110748

**Published:** 2026-06-12

**Authors:** Samar A Bou Jaoude, Nawal M El-Baba, Carina Mehanna Zogheib, Carla E Zogheib, Maha H Daou

**Affiliations:** 1 Department of Oral Medicine and Radiology, Saint Joseph University of Beirut, Beirut, LBN; 2 Department of Restorative Dentistry, Saint Joseph University of Beirut, Beirut, LBN; 3 Department of Endodontics, Saint Joseph University of Beirut, Beirut, LBN; 4 Department of Pediatric Dentistry, Saint Joseph University of Beirut, Beirut, LBN

**Keywords:** bleeding disorders, congenital heart disease, dental management, noonan syndrome, oral manifestations

## Abstract

Noonan syndrome (NS) is a rare autosomal dominant disorder characterized by distinctive craniofacial features, congenital heart disease, bleeding disorders, and variable cognitive impairment. Oral manifestations may include malocclusion, delayed tooth eruption, enamel defects, and dental anomalies. This report describes the multidisciplinary management of a 34-year-old man with NS presenting with dental trauma, unaesthetic anterior teeth, and functional concerns. Management involved surgical extractions, endodontic treatment, and prosthetic rehabilitation, coordinated with cardiology and hematology teams to minimize systemic risks. Individualized treatment planning, behavioral adaptations, and tailored hemostatic strategies facilitated safe dental rehabilitation. This case highlights the importance of interprofessional collaboration and risk-adapted dental care in patients with complex systemic conditions.

## Introduction

Noonan syndrome (NS), first described by pediatric cardiologist Jacqueline Noonan, is a rare autosomal dominant disorder with variable expressivity, resulting in a spectrum of clinical severity [1,2]. De novo mutations are common and are associated with advanced paternal age [3]. NS was historically misdiagnosed as Turner syndrome due to overlapping phenotypic features; however, NS affects both sexes and is associated with a normal karyotype [2].

NS is characterized by distinctive craniofacial dysmorphism, including a broad forehead, down-slanting palpebral fissures, hypertelorism, low-set posteriorly rotated ears, and a high-arched palate [4,5]. Systemic manifestations include short stature, congenital heart disease, lymphatic malformations, renal anomalies, and bleeding disorders [6]. Hematologic abnormalities often involve thrombocytopenia, platelet dysfunction, and deficiencies of coagulation factors VIII, XI, and XII [6]. Oral manifestations include malocclusion, delayed tooth eruption, hypodontia, enamel defects, and odontogenic anomalies such as odontomas [7]. Intellectual disability and behavioral challenges may complicate dental management [8].

Although systemic features of NS are well documented, reports on complex dental rehabilitation in adult patients with concurrent cardiac and bleeding disorders remain rare. Most of the literature focuses on pediatric patients or on isolated oral findings [7,9-11]. To date, no standardized protocol exists for the dental management of adult NS patients, and published cases of comprehensive multidisciplinary rehabilitation in this population remain exceptionally limited. The present case demonstrates a structured, multidisciplinary approach to safe dental management in an adult patient with NS, highlighting strategies to minimize procedural risk while restoring function and aesthetics, with a seven-year follow-up confirming long-term stability.

## Case presentation

Patient information

**Figure 1:**
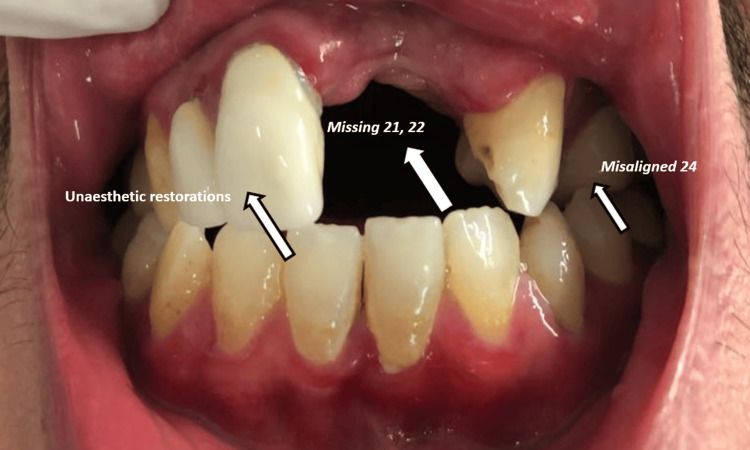
Pretreatment frontal intraoral view showing traumatic loss of maxillary left central (21) and lateral (22) incisors, unaesthetic anterior restorations, and misaligned maxillary left first premolar (24)

A 34-year-old man presented following dental trauma resulting in loss of the maxillary left central (21) and lateral (22) incisors (Figure 1). Clinical examination revealed unaesthetic crowns on contralateral anterior teeth and a misaligned maxillary left first premolar (24), causing functional discomfort.

The patient had a diagnosis of NS with congenital heart disease and mild coagulation factor deficiencies (factor VIII: 50%, factor VII: 45%). Limited communication due to intellectual disability necessitated obtaining medical history from the patient’s mother. The combination of craniofacial abnormalities, cardiac disease, and bleeding disorder posed a significant challenge for invasive dental procedures.

Clinical findings

**Figure 2:**
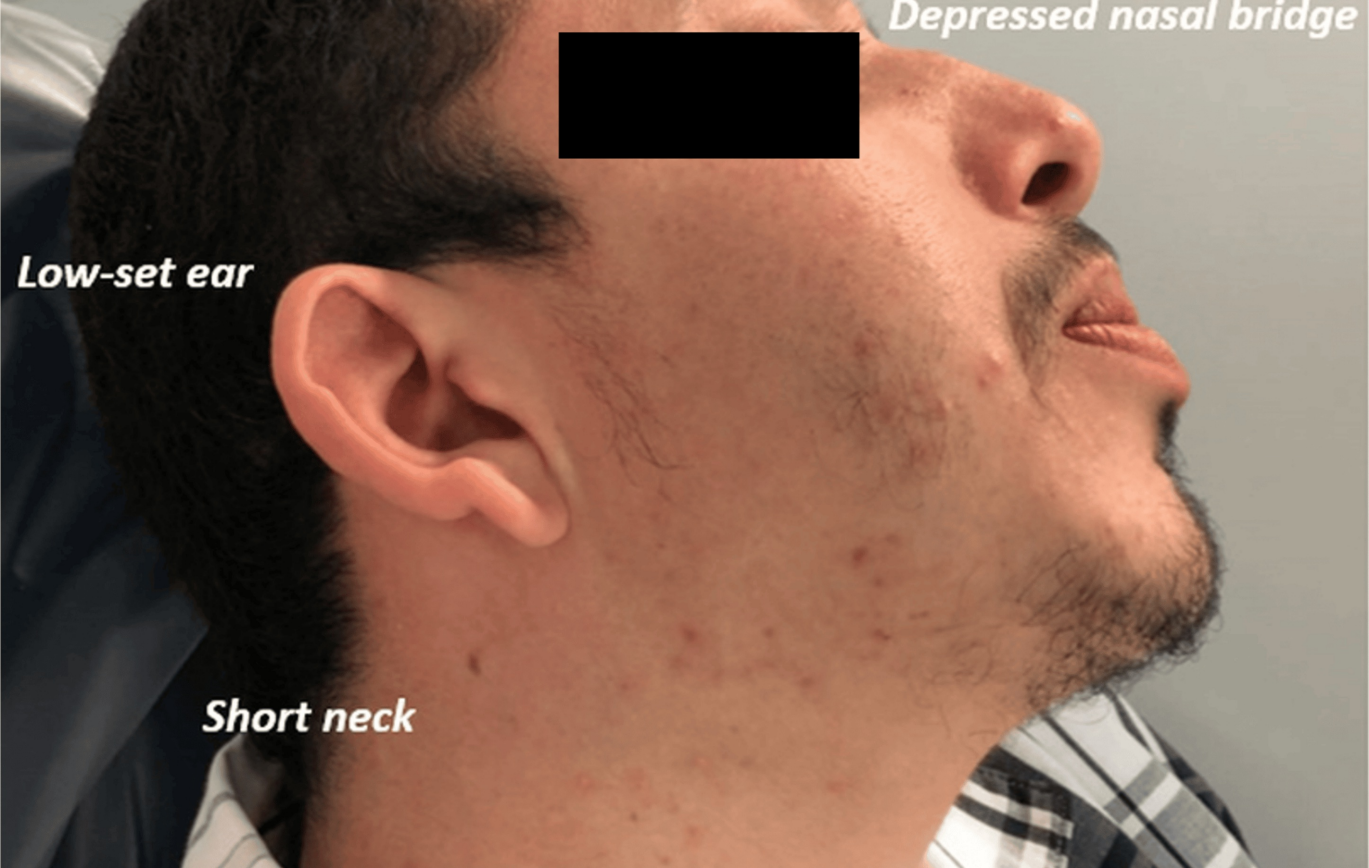
Profile view of the patient showing characteristic craniofacial features of NS including depressed nasal bridge, low-set ears, and short neck NS: Noonan syndrome Written informed consent was obtained from the patient for the publication of his clinical information and images in this open-access journal.

**Figure 3:**
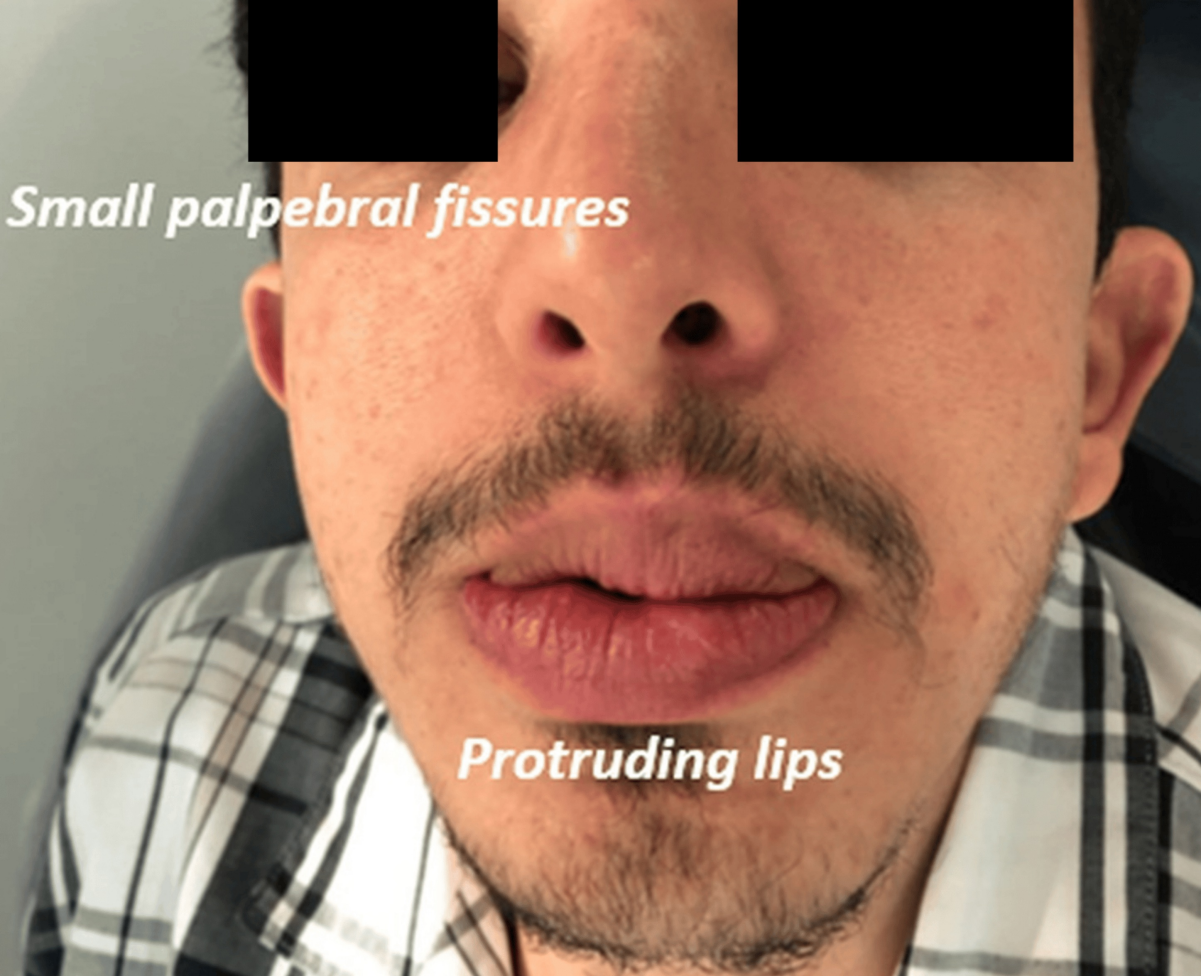
Extraoral photographs (front view) showing characteristic craniofacial features of NS including protruding lips, small palpebral fissures, and facial asymmetry NS: Noonan syndrome Written informed consent was obtained from the patient for the publication of his clinical information and images in this open-access journal.

**Figure 4:**
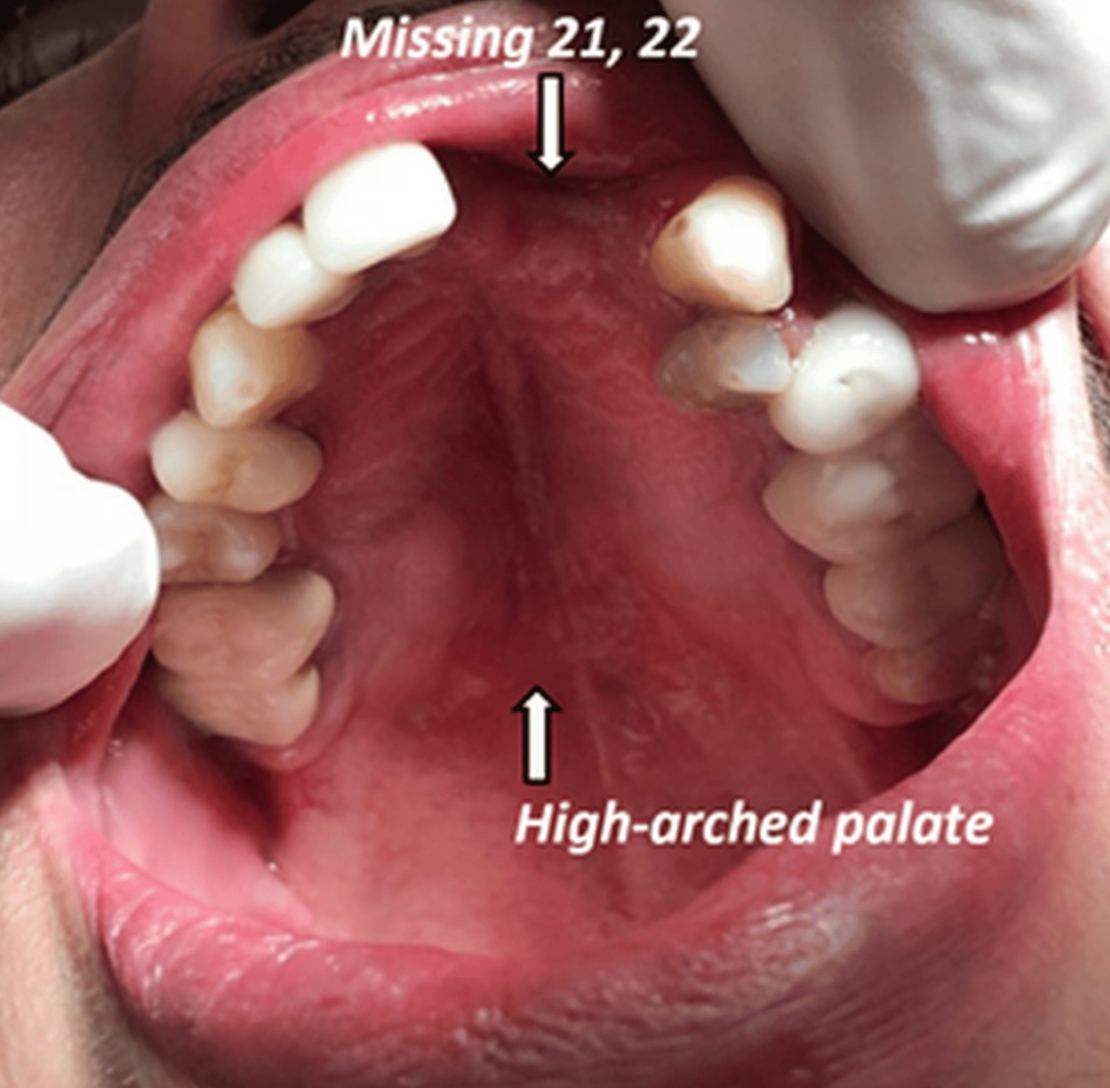
Pretreatment intraoral palatal view demonstrating the missing maxillary left central (21) and lateral (22) incisors, residual dentition, and characteristically high-arched palate associated with NS NS: Noonan syndrome

General examination revealed short stature and craniofacial features including small palpebral fissures, facial asymmetry, a depressed nasal bridge, protruding lips, low-set, posteriorly rotated ears, a short neck, ligamentous hyperlaxity, and scoliosis (Figures 2-3). Intraoral examination showed a high-arched palate (Figure 4) and a prognathic mandible.

Diagnostic assessment

Challenges included difficulty obtaining radiographs due to a high-arched palate, limited cooperation, and an increased risk of bleeding. Diagnosis of NS was previously established. Hematologic abnormalities in NS arise from dysregulation of the RAS/MAPK signaling pathway, which affects hematopoietic and coagulation processes, and may phenotypically overlap with von Willebrand disease. Preoperative hematological assessment confirmed reduced coagulation factor levels (factor VIII: 50%, factor VII: 45%), which guided perioperative management in consultation with the hematology team. Antibiotic prophylaxis (amoxicillin 2 g orally, one hour pre-procedure) was administered per current guidelines for patients at elevated risk of infective endocarditis.

Therapeutic intervention

Surgical Phase

**Figure 5:**
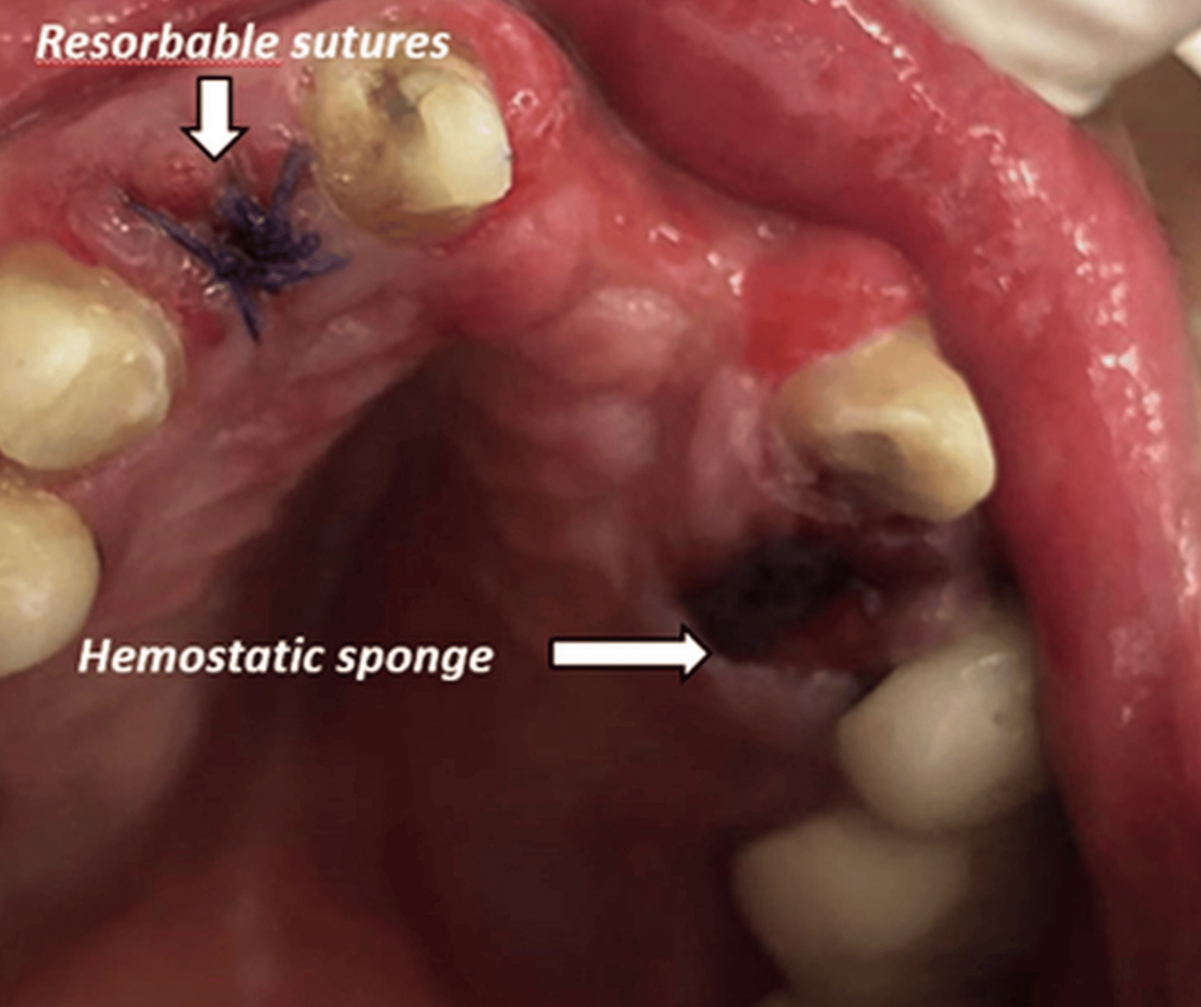
Hemostasis achieved with hemostatic sponges and resorbable suture

Non-restorable teeth (24 and 12) were extracted after factor VIII replacement. Local hemostasis was achieved using hemostatic sponges and resorbable sutures (Figure 5).

Endodontic Phase

Root canal treatment was performed on teeth 11, 13, and 23, with avoidance of over-instrumentation to minimize bleeding.

Prosthetic Phase

**Figure 6:**
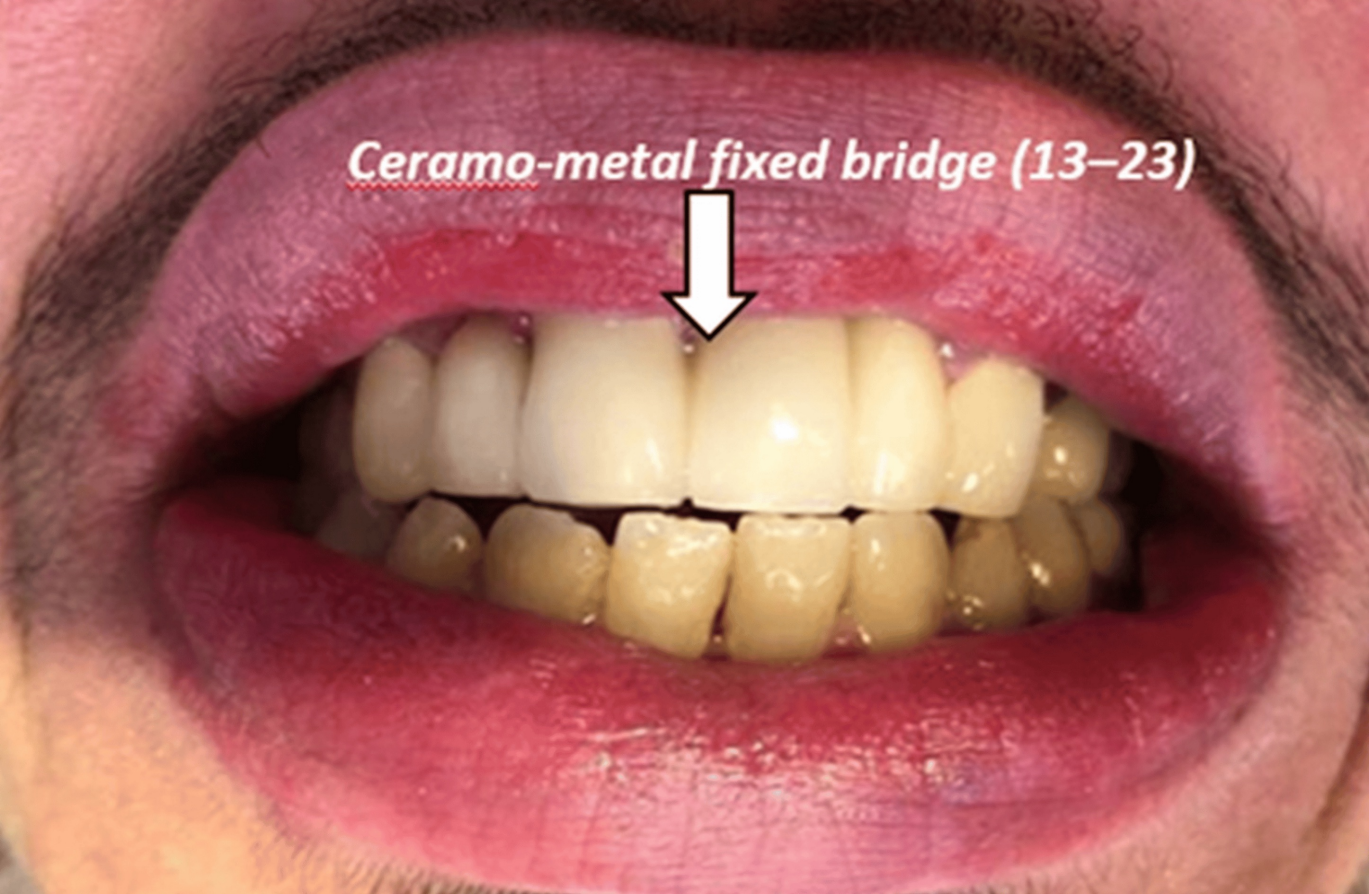
Final ceramo-metal bridge (13-23) restoring anterior function and aesthetics

A ceramo-metal fixed bridge (13-23) restored function and aesthetics. Hemostasis was maintained using a hemostatic agent; retraction cords were avoided, and a two-step impression technique was employed (Figure 6).

Follow-up and outcomes

**Figure 7:**
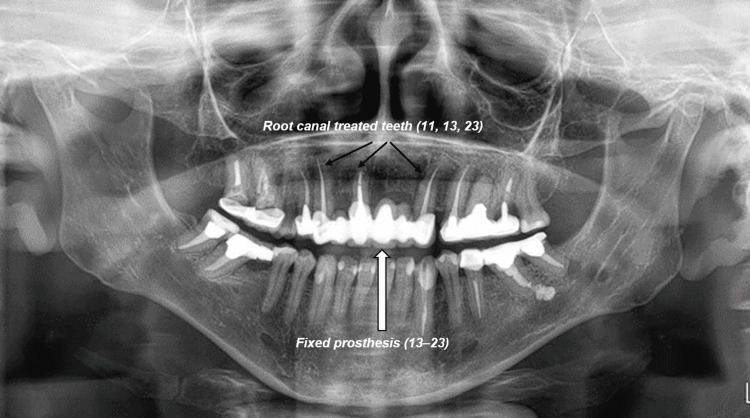
Panoramic radiograph at seven-year follow-up showing stable ceramo-metal bridge, intact alveolar bone levels, and no signs of periapical pathology or prosthetic failure

All procedures were completed successfully without complications. The patient tolerated interventions well, with improved comfort and satisfaction. Seven-year follow-up confirmed stable prosthetic rehabilitation, satisfactory oral hygiene, and long-term treatment stability (Figure 7). The overall treatment timeline, from initial presentation to seven-year follow-up, is summarized in Table 1.

**Table 1:** Chronological treatment timeline Multidisciplinary coordination with cardiology and hematology was maintained throughout all phases of invasive treatment.

Phase	Stage	Key actions
Initial assessment	Presentation	Dental trauma reported; loss of maxillary left central (21) and lateral (22) incisors noted; unaesthetic anterior crowns and misaligned premolar (24) identified
	Medical history	History obtained from caregiver due to intellectual disability; diagnosis of NS with congenital heart disease and coagulation factor deficiencies confirmed
	Multidisciplinary consultation	Cardiology and hematology referrals initiated; antibiotic prophylaxis and factor VIII replacement protocol established
Surgical phase	Extractions	Non-restorable teeth (24 and 12) extracted under factor VIII replacement coverage; local hemostasis achieved with hemostatic sponges and resorbable sutures
Endodontic phase	Root canal treatment	Root canal treatment performed on teeth 11, 13, and 23; conservative instrumentation to minimize bleeding risk
Prosthetic phase	Fixed prosthesis	Ceramo-metal fixed bridge (13-23) fabricated and delivered; hemostatic agent used; retraction cords avoided; two-step impression technique employed
Follow-up	Seven-year review	Panoramic radiograph obtained; stable prosthetic rehabilitation confirmed; satisfactory oral hygiene maintained; no complications recorded

## Discussion

NS presents significant challenges in dental care due to the frequent coexistence of craniofacial anomalies, congenital heart disease, bleeding disorders, and neurocognitive impairment. These factors increase the risk of complications, particularly during invasive dental procedures. The present case demonstrates that comprehensive dental rehabilitation can be safely achieved in an adult patient with NS through careful planning and multidisciplinary collaboration. A key strength of this case was the coordinated management with cardiology and hematology specialists, enabling invasive procedures despite underlying cardiac disease and coagulation factor deficiencies. Bleeding abnormalities are well documented in NS and may include platelet dysfunction and deficiencies of coagulation factors VIII, XI, and XII, which are not always clinically apparent before surgical intervention [6]. Tsai et al. [12] reported a severe sublingual hematoma following mandibular grafting in a patient with NS, emphasizing the possible severity of hemorrhagic complications when systemic risks are underestimated. In contrast, the present case shows that individualized hematologic assessment, perioperative optimization, and local hemostatic measures can effectively minimize these risks.

Reports of comprehensive dental rehabilitation in adult patients with NS remain exceptionally scarce in the literature, making direct comparison challenging. Tsai et al. [12] described a life-threatening sublingual hematoma following mandibular block grafting in an adult NS patient, attributable to underestimated coagulopathy, underscoring the severity of hemorrhagic complications when systemic risk factors are not fully addressed preoperatively. Sharma and MacFadyen [13] described dental management in a pediatric NS patient, focusing on behavioral challenges and preventive care, but did not address complex surgical or prosthetic rehabilitation. Similarly, Lutz et al. [7] and Gürsoy et al. [14] contributed valuable data on the oral and craniofacial phenotype of NS in predominantly pediatric cohorts. However, they did not describe multistage adult dental rehabilitation or perioperative management strategies. Esmaeelpour et al. [15] reported on dental management in NS, emphasizing individualized strategies, although the case did not involve the same degree of surgical, endodontic, and prosthetic complexity as in the present report. To our knowledge, the present case represents one of the few reports documenting a complete, complication-free multidisciplinary dental rehabilitation, including surgical extractions, root canal treatment, and fixed prosthetic reconstruction, in an adult patient with NS and concurrent congenital heart disease and coagulation factor deficiencies, with a seven-year follow-up confirming long-term stability (Table 2).

**Table 2:** Comparison with previously reported cases of dental management in NS NS: Noonan syndrome, RCT: root canal treatment

Study	Patient age	Systemic findings	Dental management	Perioperative management	Follow-up
Sharma and MacFadyen [13]	Pediatric	NS	Preventive care, behavioral management	Not detailed	Not reported
Lutz et al. [7]	Predominantly pediatric	NS, craniofacial anomalies	Oral/craniofacial phenotype description	Not detailed	Not reported
Gürsoy et al. [14]	Predominantly pediatric	NS, craniofacial anomalies	Oral/craniofacial phenotype description	Not detailed	Not reported
Esmaeelpour et al. [15]	Not specified	NS	Individualized dental strategies	Described	Not reported
Tsai et al. [12]	Adult	NS, coagulopathy	Mandibular block grafting	Underestimated bleeding risk	Complication reported
Present case	34 years	NS, congenital heart disease, factor VIII 50%, factor VII 45%	Surgical extractions, RCT, ceramo-metal fixed bridge	Cardiology and hematology consultation, factor VIII replacement, antibiotic prophylaxis	7 years, complication-free

Most published dental reports on NS focus on pediatric patients or isolated oral findings. Behavioral and cognitive factors are important considerations in dental management for patients with NS. Perrino et al. [8] reported increased rates of anxiety and cognitive impairment, which may negatively affect patient cooperation. Esmaeelpour et al. [15] further emphasized the need for individualized behavioral strategies in such patients. In this case, adapted communication, behavioral management, and caregiver involvement enabled successful completion of complex dental procedures under local anesthesia, thereby avoiding the additional risks associated with sedation or general anesthesia in a patient with cardiac and hematologic comorbidities.

Overall, this case supports existing evidence that NS requires individualized, risk-adapted dental management and extends current knowledge by demonstrating a structured multidisciplinary approach in an adult patient. The absence of intraoperative and postoperative complications, along with the successful restoration of oral function and aesthetics, highlights the importance of thorough preoperative assessment, interprofessional collaboration, and appropriate modifications to dental techniques.

Clinical implications

Patients with NS should undergo early and regular dental evaluation. Safe management requires collaboration with medical specialists, careful assessment of cardiac and bleeding risks, and adaptation of dental procedures to minimize complications. With appropriate planning, favorable functional and aesthetic outcomes can be achieved, even in complex cases.

## Conclusions

This case report demonstrates that comprehensive dental rehabilitation in an adult patient with NS is achievable when guided by thorough preoperative assessment, individualized risk management, and close multidisciplinary collaboration. While the existing literature predominantly focuses on pediatric cases, reports of complex dental rehabilitation in adult patients remain scarce, making this case a valuable contribution to the field. The seven-year complication-free follow-up highlights the effectiveness of coordinated care involving cardiology and hematology specialists, alongside adapted behavioral strategies and appropriate modifications of dental techniques. While the findings from this single case cannot be generalized to all patients with NS, they suggest that individualized interdisciplinary protocols are essential for optimizing long-term oral health outcomes in this population throughout adulthood. Larger prospective studies are needed to confirm these findings and develop standardized clinical guidelines. Early multidisciplinary assessment and individualized dental planning remain essential for the safe and effective management of patients with NS.
